# Deep vein thrombosis in mice is regulated by platelet HMGB1 through release of neutrophil-extracellular traps and DNA

**DOI:** 10.1038/s41598-018-20479-x

**Published:** 2018-02-01

**Authors:** Mitchell R. Dyer, Qiwei Chen, Shannon Haldeman, Hamza Yazdani, Rosemary Hoffman, Patricia Loughran, Allan Tsung, Brian S. Zuckerbraun, Richard L. Simmons, Matthew D. Neal

**Affiliations:** 1 Department of Surgery, University of Pittsburgh Medical Center, Pittsburgh, PA USA; 2 Center for Biological Imaging, University of Pittsburgh, Pittsburgh, PA USA

## Abstract

Venous thromboembolic (VTE) disease, consisting of deep venous thrombosis (DVT) and pulmonary embolism (PE) is a leading cause of morbidity and mortality. Current prophylactic measures are insufficient to prevent all occurrence in part due to an incomplete understanding of the underlying pathophysiology. Mounting evidence describes interplay between activation of the innate immune system and thrombus development. Recent work has demonstrated that platelet release of HMGB1 leads to increased microvascular complications following injury. Additionally, platelet HMGB1 was found to enhance DVT and increase the formation of neutrophil extracellular traps (NETs), although the role of HMGB1 induced NET release in thrombosis remains unexplored. Utilizing a transgenic mouse lacking HMGB1 specifically from platelets and megakaryocytes we now demonstrate the specific role of platelet-derived HMGB1 in acute and subacute/chronic venous thrombosis. Platelets account for the majority of circulating HMGB1 and HMGB1 deposition within the developing clot. The pro-thrombotic effect of platelet-derived HMGB1 is mediated through enhanced neutrophil recruitment, NET formation and specifically release of extracellular DNA during NET formation. Taken together, these data suggest that platelet HMGB1 mediated NET release is a primary regulator of DVT formation in mice.

## Introduction

Deep vein thrombosis (DVT) and pulmonary embolism, collectively referred to as venous thromboembolic disease (VTE), is a common thrombotic disease^1,2^. Prevention, as well as treatment of VTE disease, is achieved with anticoagulation through either heparins, vitamin K antagonist, or novel oral anticoagulants. However, even with proper prophylaxis and treatment these measures are insufficient to prevent events and recurrences as well as come with an increased risk of bleeding^2^.

VTE disease is a multifactorial process with an underlying pathophysiology that is incompletely understood. Increasing evidence suggests there is interplay between innate immune activation, sterile inflammation, and thrombus development^3–7^. High-mobility group box 1 (HMGB1), a characteristic protein released following innate immune activation has been found to be elevated in states of abnormal coagulation^3,8–10^. We have recently demonstrated that release of HMGB1 from platelets regulates inflammation and microvascular thrombosis after injury^3^. Further evidence of the important role of innate immune activation in thrombus development demonstrated a role for HMGB1 from platelets in DVT formation via recruitment of monocytes, facilitating neutrophil extracellular trap (NET) development^3,4,11–17^.

Utilizing a transgenic mouse line lacking HMGB1 from platelets and megakaryocytes, we now show that platelets are the major source of HMGB1 both in thrombus and in circulation in an acute murine model of DVT. Ablation of HMGB1 from platelets and megakaryocytes decreases thrombus burden in both the acute and subacute/chronic settings of DVT. Strikingly, we have observed the prothrombotic effects of platelet-derived HMGB1 are dependent upon increased neutrophil recruitment to the thrombus with release of neutrophil extracellular traps (NETs) and specifically, DNA. Here we present further evidence for the interplay between platelet innate immune signaling and thrombosis in DVT.

## Materials and Methods

### Animals

Mice lacking HMGB1 specifically from platelets and megakaryocytes were generated utilizing *Cre-lox* technology as previously described^3^. Briefly, *Cre-Pf4* mice were crossed with floxed *HMGB1* (*HMGB1 Flox*) mice with offspring resulting in mice lacking HMGB1 specifically from platelets and megakaryocytes (*HMGB1 Pf4*), confirmed by immunofluorescence staining and western blot analysis of isolated platelets. C57BL/6J mice were purchased from Jackson Laboratories. Mice were housed in accordance with University of Pittsburgh (Pittsburgh, PA, USA) and National Institutes of Health (NIH; Bethesda, MD, USA) animal care guidelines. All animal experiments were approved and conducted in accordance with the guidelines set forth by the Animal Research and Care Committee at the University of Pittsburgh.

### Acute and subacute/chronic deep venous thrombosis (DVT) model

*HMGB1 Pf4, HMGB1 Flox*, and C57BL/6J male mice, littermates, age 8–12 weeks, were utilized in all experiments. DVT was induced using a validated model of venous stasis, inferior vena cava (IVC) ligation^18^. Briefly, mice were anesthetized, underwent a midline laparotomy, the IVC is ligated completely with 6-0 nonabsorbable suture at the level of the renal veins, all visible side branches are ligated with 6-0 nonabsorbable sutures as well. Mice were then allowed to recover and later were sacrificed at 24 h (acute model) or 7 days (subacute/chronic) following IVC ligation. Thrombi, which included the thrombus and vessel wall, were excised and weighed immediately. Clots were processed for protein harvest or immunofluorescent (IF) staining. Where noted mice received recombinant HMGB1 (1 μg/g body weight, R&D Systems), the PAD4 inhibitor GSK199 (30 mg/kg, Cayman Chemical), or DNase I (100 units, ThermoFischer) via tail vein injection 30 min prior to IVC ligation.

### Measurement of circulating HMGB1 and P-Selectin levels

Blood was collected via cardiac puncture in heparin coated syringes at the time of sacrifice and centrifuged at 2000 g for 15 min to obtain plasma. Plasma levels of HMGB1 were measured via an enzyme-linked immunoassay (ELISA) (IBL International ST51011, Hamburg, Germany). Plasma levels of soluble P-Selectin were measured via ELISA (Thermo Scientific, EMSELP).

### Western blot analysis

Protein was harvested from IVC clots as previously described^3^ and subjected to western blot analysis using the following primary antibodies: anti-HMGB1 polyclonal (1 ug/ml, Rabbit IgG; ab18256, Abcam) and anti-Actin polyclonal (1:1000, Rabbit IgG; ab18101, Abcam) and the following secondary antibody Goat anti-Rabbit IgG (ab’)2 (1:20,000; 31641, Thermo Scientific).

### Immunofluorescence staining for NETs

Clots were fixed in 2% paraformaldehyde for 2 h and then switched to 30% sucrose 24 h. The clot was then frozen and 6 µm sections were blocked in 20% normal goat serum. The samples were then incubated overnight with a combination of the following primary antibodies: anti-CD41 (5 μg/ml, rat IgG; Abcam, ab33661); citH3 (5 μg/ml, rabbit IgG; Abcam ab5103); Ly6G-APC (1:1000, BD Pharmogen 560599) or HMGB1 (2 μg/ml, rabbit IgG; Abcam 18256). Sections were then incubated with Alexa 488-conjugated F-actin phalloidin (1:500, Invitrogen, San Diego, CA, USA) in the presence of the following secondary antibodies depending on the primary antibody pairing: Cy5–conjugated goat anti-rat IgG (1:1000, for anti-CD41 antibody, Jackson Immunoresearch 112-165-167); 488-conjugated goat anti-rat IgG (1:500 for CD41 antibody, Molecular Probes, A11006); Cy3-conjugated goat anti-rabbit IgG (1:1000, for anti-HMGB1 antibody, Jackson Immunoresearch 111-165-003) for 1 h. A Hoeschst nuclear stain was applied for 30 s and slides were prepared for imaging. Imaging conditions were maintained at identical settings within each antibody-labeling experiment with original gating performed using the negative control. Large area images in X and Y were obtained thru the Z plane and are presented as a mean intensity projection of Z using a Nikon A1 confocal microscope (purchased with 1S10OD019973-01 awarded to Dr. Simon C. Watkins). Quantification was performed using NIS Elements Software (Nikon). NETs were quantified by the presence of extracellular Histone 3 staining co-localized with Ly6G staining^3,19^.

### Neutrophil isolation and *in vitro* NET formation

Mouse neutrophils were isolated from the bone marrow of tibias and femurs from *HMGB1 Flox* and *HMGB1 Pf4* mice. Neutrophils were sorted on a BD Aria Plus high-speed sorter after incubation with APC-conjugated anti-mouse Ly6G antibody and APC-Cy7 CD11b (BD Biosciences). Neutrophils were plated and allowed to adhere to coated plates for 1 h before stimulation with recombinant HMGB1 (1 μg/ml) or LPS (10 μg/ml). Neutrophils were then fixed in 2% paraformaldehyde and incubated with antibodies for CitH3 (1:50, Abcam, ab52013) and a DAPI nuclear stain. Confocal images were acquired using Olympus Fluoview 1000 microscope. NETs were identified by the presence of extracellular CitH3.

### Flow cytometry

A single cell suspension was prepared from spleen and red blood cells were lysed. To identify neutrophils, cells were incubated with anti-CD11b (eBioscience, clone M1/70) to label monocytes and anti-CD11b plus anti-Ly6G (BD Biosciences, clone 1A8) to label granulocytes. Cells were washed, fixed and permeabilized with Foxp3 Fixation and Permeabilization kit, eBioscience) followed by incubation with anti-HMGB1 (Biolegend, clone3E8). Data was acquired with a Becton Dickinson LSRII instrument.

### Statistics

All data are presented as mean ± standard deviation for *n* ≥ 3 unless stated otherwise in the figure legends. Statistical significance was determined with two-tailed Student’s *t* Test using GraphPad software with *p* < 0.05 considered significant.

### Data availability

The datasets generated during and/or analyzed during the current study are available from the corresponding author on reasonable request.

## Results and Discussion

### HMGB1 is elevated following DVT and contributes to thrombus formation

**Figure 1:**
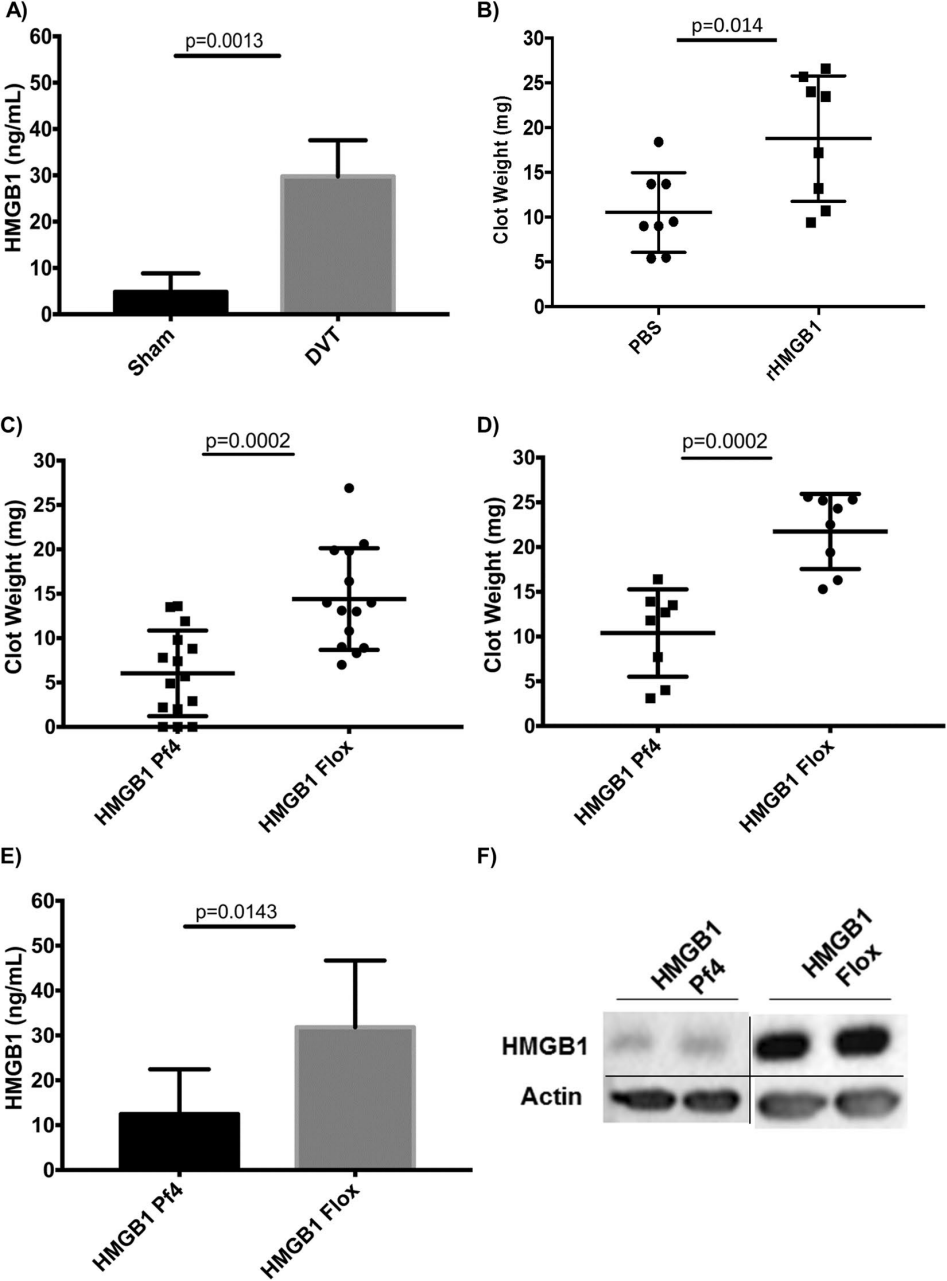
Platelet-derived HMGB1 promotes DVT formation. (**A**) HMGB1 levels are significantly elevated following development of acute DVT as assessed by measurement of plasma levels via ELISA in mice subjected to IVC ligation compared to a sham operation. (**B**) C57BL/6J mice treated with rHMGB1 had significantly higher thrombus burden, as assessed by clot weight, at 24 h compared to vehicle control. (**C**) In the acute setting of DVT (24 h) *HMGB1 Pf4* have significantly less clot formation compared to *HMGB1 Flox* mice (**D**) At one week, a subacute/chronic time point in murine DVT, the effect of platelet-HMGB1 persists in the remodeling clot as *HMGB1 Pf4* mice continue to have a lower thrombus burden. (**E**) Following acute DVT the majority of HMGB1 in circulation appears to be derived from platelets as demonstrated by significantly lower plasma levels of HMGB1 as measured via ELISA in *HMGB1 Pf4* compared to *HMGB1 Flox*. (**F**) Western blot analysis of acute DVT clots from *HMGB1 Pf4* and *HMGB1 Flox* mice reveal the HMGB1 deposition within the clot is nearly exclusively from platelets. Western blot are representative images from full-length gel. Data is presented as mean ± SD with all experiments n > 3 mice per group. (**A**–**E**) two-way Student’s *t* Test.

Extracellular HMGB1 has been implicated in development of thrombotic complications in coronary disease, disseminated intravascular coagulation, trauma, and DVT^3,4,8,20^. To further investigate the role of circulating HMGB1 in venous thrombosis development we utilized a validated model of IVC ligation to induce DVT development. Twenty-four hours following IVC ligation, circulating levels of HMGB1 are significantly higher compared to sham operated mice (4.84 ± 1.97 vs 29.77 ± 3.89 ng/ml, *p* = 0.0013, Fig. 1A), suggesting a potential role for HMGB1 in the pathogenesis of the disease. HMGB1 has been shown to be upregulated in numerous disease states that are complicated by a high incidence of VTE, including trauma, sepsis, and various cancers^21–23^. With these observations, we next tested the hypothesis that administration of recombinant HMGB1 would worsen the thrombus burden in DVT. Given that prior work has shown the redox state of HMGB1 regulates thrombosis, we chose a recombinant HMGB1 that we have previously validated to enhance clot formation in *ex vivo* induced thrombosis to confirm the *in vivo* pro-thrombotic effect of HMGB1 in DVT^3,4^. At 24 h mice pre-treated with recombinant HMGB1 via intravenous delivery had a significantly higher thrombus burden compared to vehicle control (10.5 ± 4.4 vs. 18.8 ± 7 mg, *p* = 0.014, Fig. 1B). Together, these findings suggest that HMGB1 is released into circulation during development of DVT and contributes to thrombus formation.

### HMGB1 release from platelets promotes acute and subacute/chronic DVT formation

HMGB1 is ubiquitously expressed and released from multiple cell-types following innate immune activation. Following identification of elevated HMGB1 levels during DVT development we sought to identify the cellular source of HMGB1 during DVT. There is increasing literature linking innate immune activation to platelet activation and action. Specifically, HMGB1 has been shown to bind and activate platelets and release of HMGB1 from platelets has been shown to be important in thrombus development^3,4,20^. Seeking to examine the specific role of platelet derived HMGB1 in DVT, mice lacking HMGB1 from platelets and megakaryocytes (*HMGB1 Pf4*) were subjected to IVC ligation and thrombus burden was quantified. At 24 h, *HMGB1 Pf4* have a significantly decreased clot burden compared to *HMGB1 Flox* control as assessed by clot weight (6 ± 4.8 vs. 14.4 ± 5.7 mg, *p* = 0.0002, Fig. 1C). Strikingly, the effect of HMGB1 release by platelets persists into the subacute/chronic phase of DVT development, as at 1-week *HMGB1 Pf4* mice have a sustained decrease in thrombus burden compared to *HMGB1 Flox* (10.4 ± 4.8 vs. 21.7 ± 4.2 mg, *p* = 0.0002, Fig. 1D). Interestingly, platelets not only appear to be the major source of HMGB1 within the clot in the acute phase of DVT, as previously suggested by others^4^, but also the major source in the circulation as well (Fig. 1E,F). These results were surprising given that HMGB1 has been shown in other models of sterile inflammation to be rapidly elevated and then cleared after injury, and the mechanism of sustained activity of platelet HMGB1 remains unknown^21^. One potential hypothesis is ongoing platelet activation and turnover during thrombus maturation in DVT, although this remains to be tested. The recruitment of leukocytes to the thrombus site has been shown to be important in models of DVT^4,6,13,18^. To confirm the specificity of HMGB1 deletion from megakaryocytes and platelets in our murine lineage, the levels of HMGB1 were determined in granulocytes and monocytes from the spleens of *HMGB1 Pf4* and *HMGB1 Flox* mice. As shown in (Figure S1), flow cytometry revealed no significant difference in HMGB1 expression on these immune cells from the different strains. These observations further support the unique role of platelet and megakaryocyte derived HMGB1 in thrombosis. Taken together, these data reflect the critical importance of platelet activation and release of HMGB1 in DVT, both in acute and chronic settings.

### HMGB1 release from platelets enhances neutrophil recruitment and promotes NET formation which regulates thrombosis

Neutrophils can be activated to undergo a process by which they extrude their nuclear contents to the extracellular space to produce neutrophil extracellular traps (NETs). The main contents of NETs include extracellular DNA, histones, and other granular protein contents. This process, originally described as an anti-bacterial mechanism, has gained attention in the thrombosis literature with accumulating evidence demonstrating NETs have a pro-thrombotic effect^12–17,24,25^. Platelets and neutrophils interact to regulate NET formation and we and others have recently examined the role of platelet HMGB1 in NET signaling in various models of micro and macrovascular thrombosis^3,4,11^.

**Figure 2:**
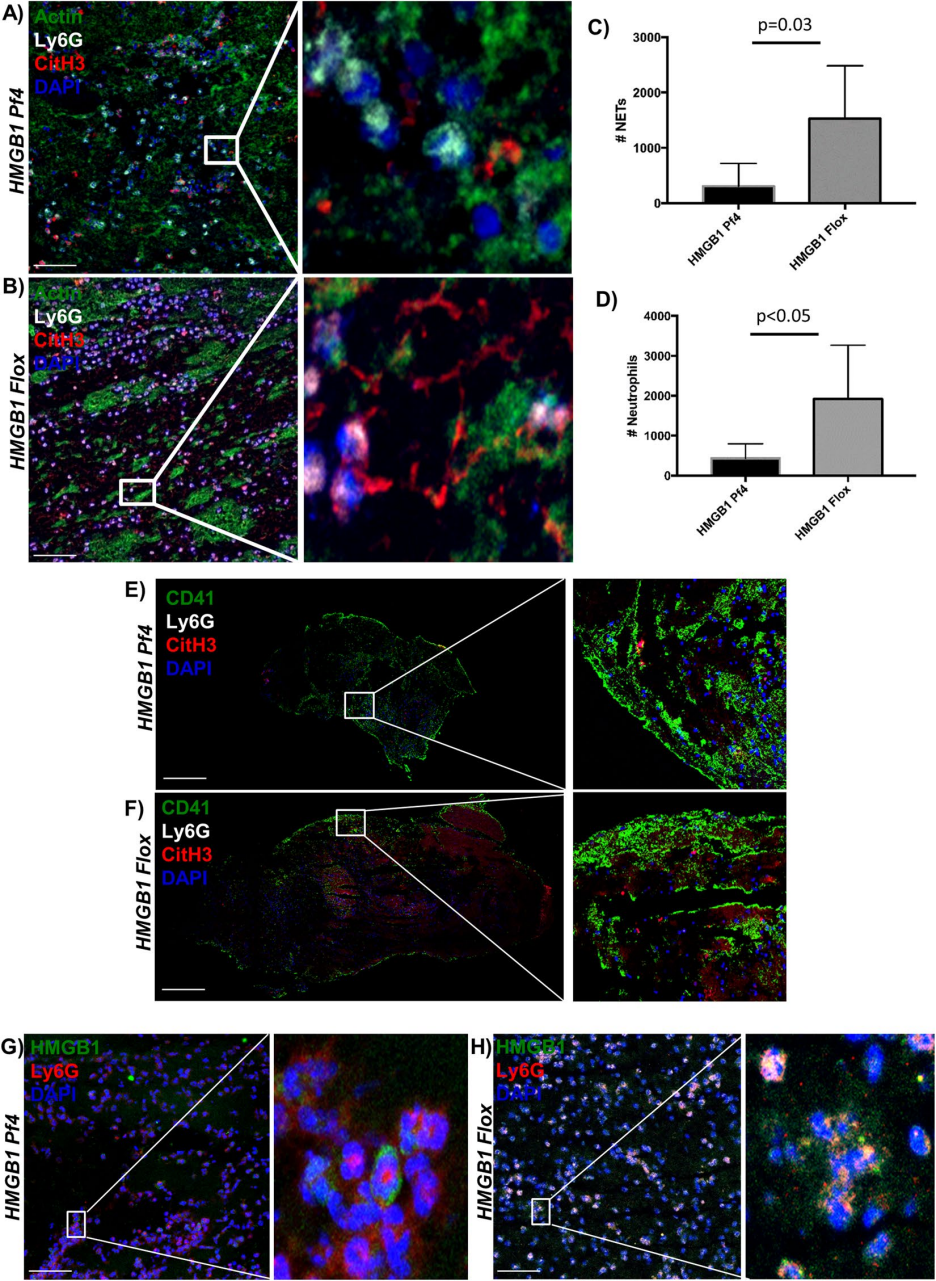
Release of HMGB1 by platelets mediates thrombosis via neutrophil recruitment and NET formation. (**A**,**B**) Immunofluorescence (IF) staining of an IVC thrombus from an *HMGB1 Pf4* mouse demonstrates small amounts of NET formation as demonstrated by extracellular citrullinated Histone-3 associated with a neutrophil compared to *HMGB1 Flox* thrombus at 24 h, which demonstrates a much greater amount of NET formation by IF staining for co-localization of CitH3 and Ly6G. Scale bar: 50 μm (**C**) Quantification of the number of NETs present in each clot. (**D**) Quantification of the number of neutrophils present within the clot. (**E**,**F**) IF imaging of thrombi from *HMGB1 Pf4* and *HMGB1 Flox* demonstrate platelet aggregation around the developing thrombus, which co-localizes with neutrophils and NETs. Scale bar: 500 μm (**G**,**H**) IF imaging of thrombi from acute DVT demonstrates co-localization of HMGB1 with neutrophils, which is increased in *HMGB1 Flox* mice. Scale Bar: 50 μm. (**A**,**B**,**E**–**H**) are representative images from n > 3 mice per experiment and images presented are a stack of at least n = 10 images. Data is presented as mean ± SD with all experiments n > 3 mice per group. (**C**,**D**) two-way Student’s *t* Test.

We therefore hypothesized that release of HMGB1 via platelets would enhance NET formation and regulate thrombus burden in murine DVT. To confirm the role of HMGB1 release from platelets we performed immunofluorescence staining for NETs on clots from the acute DVT setting and found that *HMGB1 Pf4* mice formed significantly fewer NETs (NET number 307 ± 413 vs 1529 ± 954, *p* = 0.03, Fig. 2A–C) than *HMGB1 Flox*, supporting the proposed role for platelet HMGB1 in NET release in DVT. Importantly, immunostaining of isolated neutrophils from *HMGB1 Flox* and *HMGB1 Pf4* mice demonstrates intact NET production by *HMGB1 Pf4 neutrophils* stimulated *in vitro* with recombinant HMGB1 and LPS (Figure S2), suggesting that the decreased formation of NETs in clots from *HMGB1 Pf4* mice may be due to platelet deletion of HMGB1. Interestingly, we found release of HMGB1 from platelets increased neutrophil recruitment to the thrombus site (431 ± 162 *HMGB1 Pf4* vs 1918 ± 603 *HMGB1 Flox*, p < 0.05, Fig. 2D). It has been shown that platelet HMGB1 enhances monocyte recruitment to the liver and lung in a murine model of trauma and to the thrombus in DVT; however, prior work did not identify enhanced neutrophil recruitment in DVT^4,26^. The importance of the platelet-neutrophil interaction is highlighted by immunostaining of thrombi from *HMGB1 Flox* and *HMGB1 Pf4* mice that demonstrated platelet aggregation at the edges of the developing clot that was associated with neutrophils and NET formation (Fig. 2E,F). This process appears to be enhanced in *HMGB1 Flox* mice. Finally, the importance of platelet-release of HMGB1 is demonstrated in Figure G-H, where there is co-localization of HMGB1 with neutrophils, which is again increased in *HMGB1 Flox* mice compared to *HMGB1 Pf4*.

CD62P has been shown to promote NETosis and prior work has shown treatment with HMGB1 leads to increased CD62P expression on platelets. Therefore, we examined the release of soluble CD62 from platelets from *HMGB1 Flox* and *HMGB1 Pf4* mice^3,17^. At baseline there are no differences in circulating levels of CD62 (21542 pg/ml *HMGB1 Pf4* vs. 30015 pg/ml *HMGB1 Flox*, p = 0.09); however, following acute DVT, circulating levels of soluble CD62 are significantly higher in *HMGB1 Flox* as compared to *HMGB1 Pf4* mice (67545 pg/ml *HMGB1 Pf4* vs. 105806 pg/ml *HMGB1 Flox*, p = 0.033). This suggests platelet HMGB1 acts in a dual manner to: 1) recruit and directly activate neutrophils at the thrombus site and 2) to regulate CD62P surface expression and release of soluble CD62 which in turn leads to NETosis and propagation of clot formation^26,27^. Despite the observation of decreased NETs within the thrombus from mice lacking HMGB1 on platelets, the specific role of platelet-HMGB1 induced NETs in thrombosis remains incompletely explored^4^.

### Inhibition of NETosis reverses the pro-thrombotic effect of platelet-HMGB1 in acute DVT

**Figure 3:**
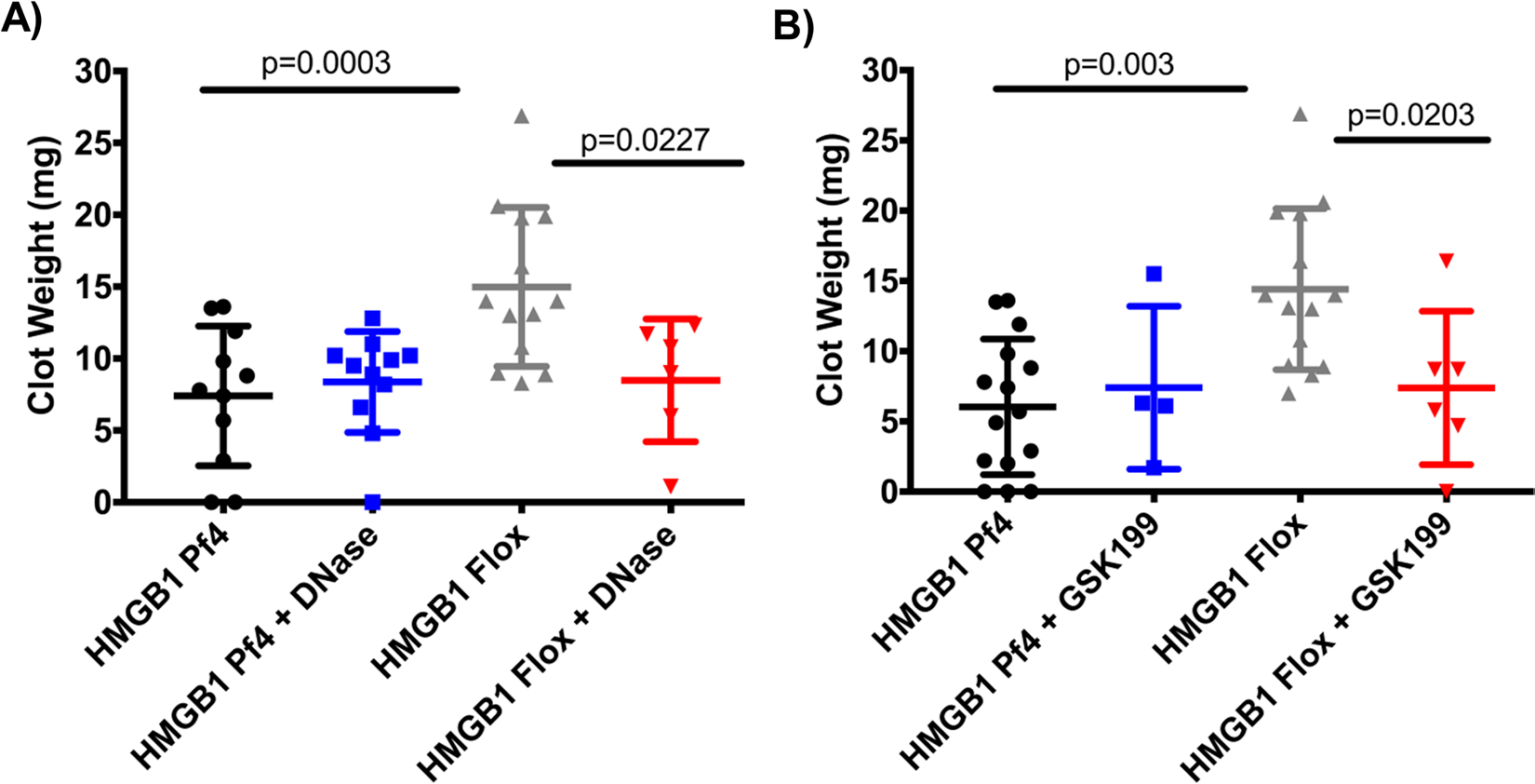
Inhibition of NETosis reverses the pro-thrombotic effect of platelet-HMGB1 in acute DVT. (**A**) Treatment with DNase, an enzyme that degrades free extracellular and circulating DNA eliminated the difference in thrombus burden between *HMGB1 Pf4* and *HMGB1 Flox* mice, suggesting the NETs exert their prothrombotic effor through release of DNA.  **(B)**  GSK199, a reversible and specific inhibitor of the PAD4 enzyme, led to a reversal of the prothrombotic effect of platelet-derived HMGB1 as assessed by clot weight at 24 h. (**A**,**B**) two-way Student’s *t* Test.

To determine whether the difference noted in NET formation between *HMGB1 Pf4* and *HMGB1 Flox* mice accounted for the difference in clot burden, we sought to inhibit the formation of NETs through blocking protein arginine deiminase 4 (PAD4), the enzyme critical in formation of NETs^14,16^. Treatment with the reversible PAD4 inhibitor, GSK199, prior to IVC ligation, led to a reversal of the pro-thrombotic effect of platelet HMGB1 (7.4 ± 2.9 vs. 8.9 ± 2 mg, p = 0.6844, Fig. 3A) with a significant reduction in NET formation (data not shown). It is currently unknown what aspect of the NET provides the prothrombotic effect but evidence suggests that DNA released into the extracellular space during NET release plays an important role^28–30^. To test the specific hypothesis that extracellular DNA mediates the pro-thrombotic tendency of platelet HMGB1 induced NETs, we pre-treated mice with DNaseI, an enzyme that catalyzes hydrolytic cleavage of DNA, 30 min prior to IVC ligation to degrade the DNA released by NETs. Importantly, treatment with DNaseI eliminated the thrombus difference between *HMGB1 Pf4* and *HMGB1 Flox* (7.9 ± 1.2 vs. 9 ± 1.3 mg, p = 0.2879, Fig. 3B). Immuno-staining revealed reduced NET formation in GSK199 treated and elimination of NETs in DNase treated mice (data not shown).

As mentioned previously, HMGB1 is ubiquitously expressed which makes it less attractive as a therapeutic target and inhibition of HMGB1 on a global scale would certainly have unknown consequences on the immune function and overall coagulation. However, the data presented here highlight the specific importance of HMGB1 released from platelets in thrombus development, specifically through neutrophil recruitment and NET formation. Alternatively, a better understanding of the mechanism and regulation of release of HMGB1 by platelets would allow for a platelet targeted therapeutic approach or targeting the end-product of platelet-HMGB1 in thrombus development, namely NET formation offers a more reliable approach.

Taken together, the data presented demonstrates an important role for HMGB1 in venous thrombosis. Specifically, HMGB1 released from platelets leads to neutrophil recruitment, activation, and NET formation, which results in enhanced thrombus formation via the pro-thrombotic effects of the extracellular DNA released during NETosis. Attenuation of platelet release of HMGB1 or HMGB1 induced NET formation could represent a promising targeted approach in VTE prophylaxis and treatment.

## Electronic supplementary material

Supplementary Information
