# Supplementary material for: Deep vein thrombosis in mice is regulated by platelet HMGB1 through release of neutrophil-extracellular traps and DNA
Source: Sci Rep. 2018 Feb 1;8:2068. doi: 10.1038/s41598-018-20479-x (PMC5794752; doi:10.1038/s41598-018-20479-x)
Supplement: Supplementary file 1 — Supplementary Information [file 41598_2018_20479_MOESM1_ESM.pdf]

Deep vein thrombosis in mice is regulated by platelet HMGB1 through release of neutrophil-extracellular traps and DNA

Authors:

Mitchell R. Dyer[1], Qiwei Chen[1], Shannon Haldeman[1], Hamza Yazdani[1], Rosemary Hoffman[1], Patricia Loughran[1,2], Allan Tsung[1], Brian S. Zuckerbraun[1], Richard L. Simmons[1], and Matthew D. Neal[1]

Supplementary Figures

Figure S1. HMGB1 expression in granulocytes and monocytes. HMGB1 expression was determined in granulocytes and monocytes from *HMGB1 Flox* and *HMGB1 Pf4* mice by flow cytometry. No difference was found in either granulocytes or monocytes between the different strains.

Figure S2. HMGB1 and LPS induce NET formation and *HMGB1 Flox* and *HMGB1 Pf4* mice. Isolated neutrophils from *HMGB1 Flox* and *HMGB1 Pf4* were treated with either recombinant HMGB1 or LPS for 4 hours to induce NET formation. IF staining for CitH3 demonstrates intact NETosis in neutrophils in response to HMGB1 from both strains of mice.

Figure S3. Platelets are the primary source of HMGB1 within the venous thrombus. Western blot analysis of HMGB1 within the venous clot from *HMGB1 Flox and HMGB1 Pf4 mice. HMGB1 deposition within the clot appears to be exclusively from platelets. Actin served as a loading control. Unmodified images.*

Figure S1.

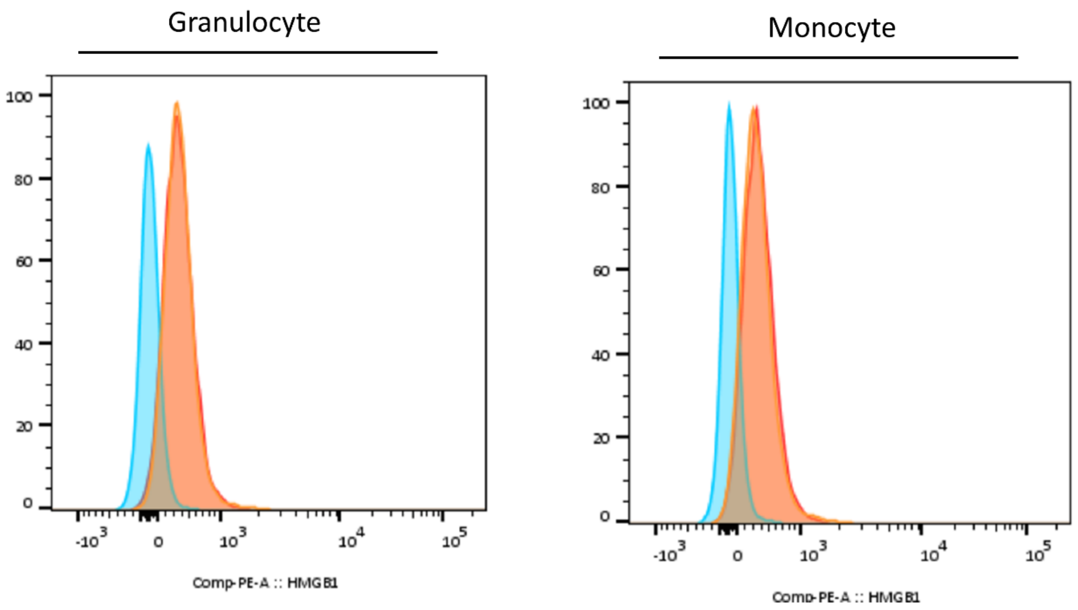

	Sample	Subset	Count
	Fluorescence Minus One (FMO)	CD11b,Ly6G	3814
	HMGB1 Pf4	CD11b,Ly6G	9361
	HMGB1 Flox	CD11b,Ly6G	7460

	Sample	Subset	Count
	Fluorescence Minus One (FMO)	CD11b	8774
	HMGB1 Pf4	CD11b	21064
	HMGB1 Flox	CD11b	16329

Figure S2.

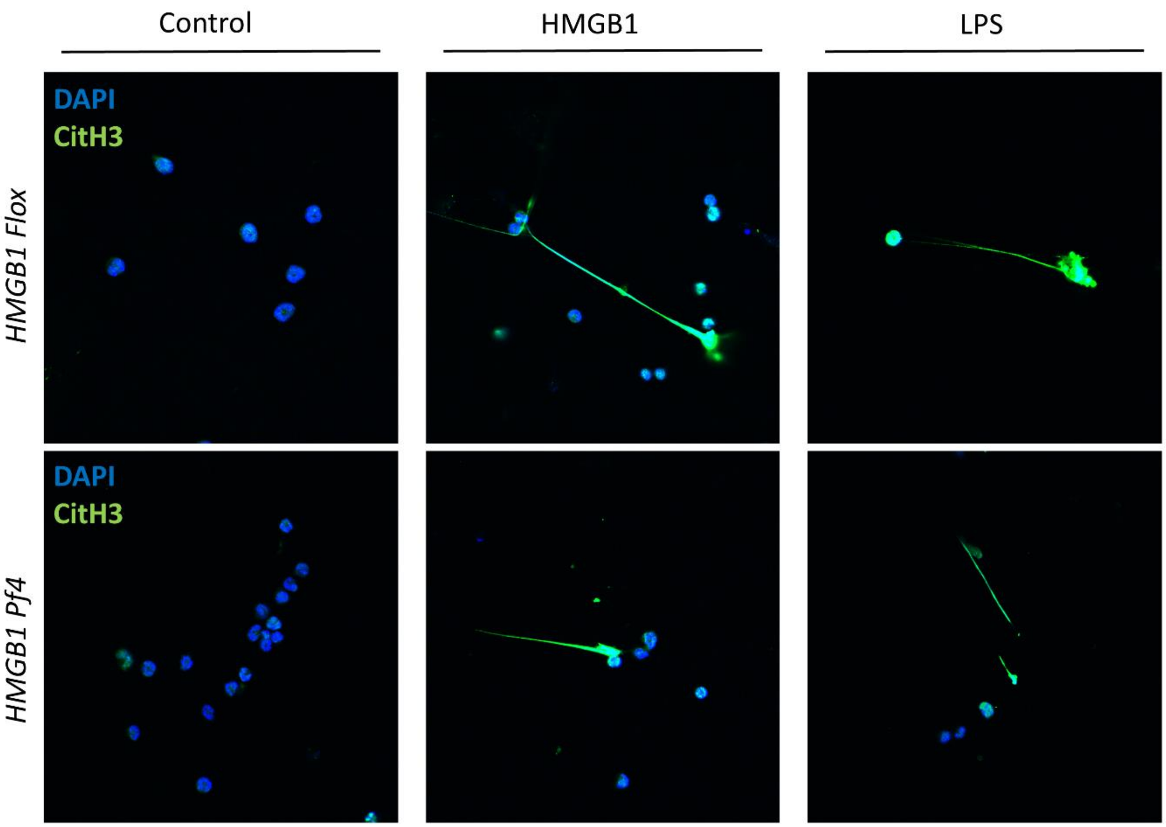

Control
HMGB1
LPS
DAPI
CitH3
HMGB1 Flox
DAPI
CitH3
HMGB1 Pf4

Figure S3.

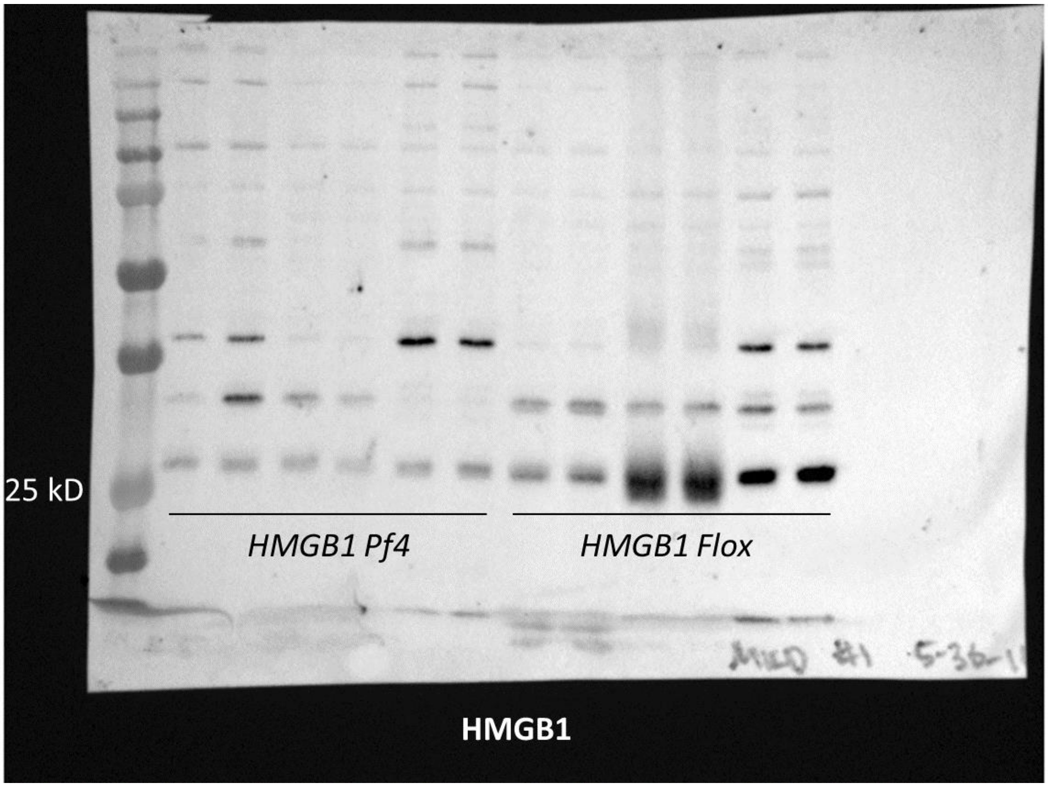
25 kD
HMGB1 Pf4
HMGB1 Flox
HMGB1

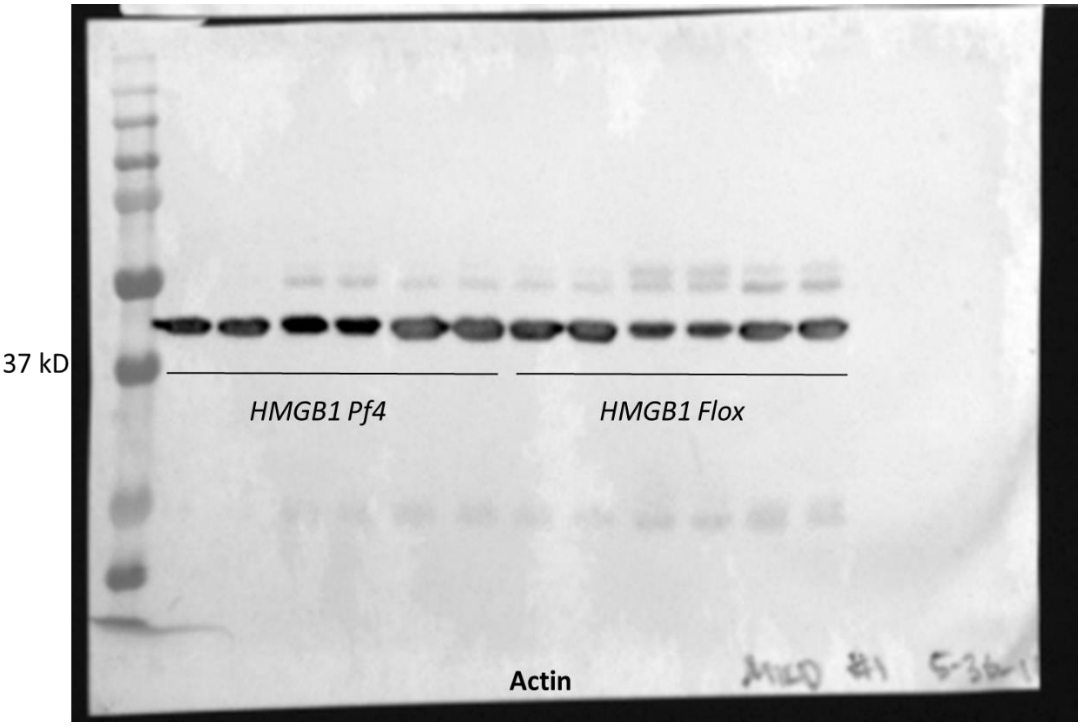
37 kD
HMGB1 Pf4
HMGB1 Flox
Actin
